# In-host co-colonization and bloodstream infection by distinct classical and hypervirulent CRKP clones harboring a homologous *bla*_KPC-2_-harboring plasmid

**DOI:** 10.3389/fcimb.2025.1683743

**Published:** 2025-12-19

**Authors:** Haixing Fang, Yueliang Chen, Ying Chen, Yan Qi, Rushuang Yan, Feng Guo

**Affiliations:** 1 Department of General Surgery, Sir Run Run Shaw Hospital, Zhejiang University School of Medicine, Hangzhou, China; 2 Department of Critical Care Medicine, Sir Run Run Shaw Hospital, Zhejiang University School of Medicine, Hangzhou, China; 3 Zhejiang Key Laboratory of Precise Diagnosis and Treatment of Abdominal Infection, Sir Run Run Shaw Hospital, School of Medicine, Zhejiang University, Hangzhou, China; 4 Medical Laboratory Center, Hangzhou Traditional Chinese Medicine (TCM) Hospital Affiliated to the Zhejiang Chinese Medical University, Hangzhou, China; 5 Zhejiang Key Laboratory of Precision Diagnosis and Therapy for Major Gynecological Diseases, Women’s Hospital, Zhejiang University School of Medicine, Hangzhou, China

**Keywords:** CRKP, co-colonization, BSI, hyper-virulence, *bla*_KPC-2_

## Abstract

Carbapenem-resistant *Klebsiella pneumoniae* (CRKP) readily colonizes clinical environments as well as the respiratory and intestinal tracts of patients and can easily cause secondary infections, posing a serious threat to infection treatment and hospital infection control. In this study, we continuously tracked CRKP strains isolated from a single patient over a total of 16 weeks—from admission to the ICU until discharge from the general ward. A total of 21 CRKP strains were obtained, including 1 bloodstream infection (BSI) isolate, 8 respiratory tract colonization isolates, and 12 intestinal colonization isolates. All isolates were evaluated for their phenotypic characteristics related to antimicrobial resistance, pathogenicity, and plasmid transfer through antimicrobial susceptibility testing, mouse infection models, and conjugation experiments. In parallel, whole-genome sequencing was performed to determine their MLST types, capsular serotypes, resistance and virulence genes, and plasmid profiles. The results showed that these isolates belonged to two distinct clones. One BSI isolate, along with one respiratory and one intestinal colonization isolate, belonged to the ST268 with capsular serotype KL20. This clone carried not only the typical hypervirulence (hv) genes *aerobactin*, *colibactin*, and *rmpA2* but also a plasmid encoding *bla*_KPC-2_, representing a classic CR-hvKP strain. Mouse infection models confirmed its high virulence. The remaining isolates belonged to the ST4496 clone, a member of the CC11 clonal complex commonly found in ICU outbreaks, with serotype KL47, exhibiting lower pathogenicity but carrying the same *bla*_KPC-2_-harboring plasmid as ST268, indicating horizontal plasmid transfer. In-host co-colonization by the distinct CRKP clones ST4496 and ST268 may have facilitated horizontal transfer of the *bla*_KPC-2_-harboring plasmid, enabling ST268 to evolve from hvKP into CR-hvKP and subsequently cause secondary BSI. This process, in which pathogen clones with different traits co-colonize and mutually promote evolutionary changes, may interfere with clinical treatment decisions and underscores the need for more intensive hospital infection surveillance.

## Introduction

Carbapenem-resistant *Klebsiella pneumoniae* (CRKP) has emerged as one of the most formidable challenges in clinical infection management, representing a critical threat to global public health. The therapeutic challenges posed by CRKP are compounded by limited treatment options, with many strains exhibiting even pan-drug-resistant (PDR) phenotypes (Chen et al., 2024). Furthermore, CRKP demonstrates a remarkable propensity for colonization of the nasopharyngeal tract and gastrointestinal system, establishing persistent reservoirs that serve as sources for subsequent infections (Sun et al., 2019; Qin et al., 2020; Du et al., 2023). The transition from colonization to infection represents a particularly dangerous clinical progression, with studies demonstrating that 64% of patients with *K. pneumoniae* infections are colonized by clonally related strains (Du et al., 2023). This rapid progression from colonization to invasive disease underscores the significant clinical threat posed by CRKP.

The molecular epidemiology of CRKP exhibits distinct regional patterns, with China being characterized by the predominance of ST11 and its related clonal complex CG11 as the dominant lineages responsible for carbapenem resistance dissemination, with some studies reporting ST11 prevalence rates exceeding 95% among clinical CRKP collections (Meng et al., 2019; Wang et al., 2022; Shi et al., 2024). Within the CG11 clonal complex, ST4496 represents a noteworthy derivative clone that has demonstrated capacity for transient hospital outbreaks, emerging as a single-allele variant of ST11 differing only in the *mdh* locus (Zhao et al., 2021). This sequence type was observed to spread within ICU settings for approximately 6 months before disappearing, with phylogenetic analysis revealing that ST4496-KL47 strains were probably derived from ST11-KL47 via intraspecies shifting (Zhao et al., 2021).

Hypervirulent *K. pneumoniae* (hvKP) has traditionally been associated with specific capsular serotypes that demonstrate enhanced pathogenic potential compared to classical *K. pneumoniae* strains. The most prominent hvKP lineages include K1 serotype strains belonging to ST23, as well as K2 serotype strains classified as ST65 and ST86 (Stanton and Wyres, 2024). These lineages have been extensively documented as causative agents of severe invasive infections, particularly pyogenic liver abscesses and community-acquired pneumonia in otherwise healthy individuals (Sohrabi et al., 2022). Beyond the classical K1 and K2 serotypes, emerging evidence indicates that other capsular types, including KL20, may contribute to hypervirulent phenotypes (Shen et al., 2020; Goncalves et al., 2025). The convergence of carbapenem resistance and hypervirulence represents one of the most alarming developments in contemporary bacterial pathogen evolution, occurring through two primary evolutionary pathways: classical CRKP strains acquiring virulence plasmids (resulting in hv-CRKP strains) and hvKP strains acquiring carbapenemase-encoding plasmids (generating CR-hvKP strains) (Han et al., 2022; Tian et al., 2022; Zhang et al., 2024). Current evidence suggests that the hv-CRKP pathway is more prevalent in clinical settings, likely due to the facilitative role of *bla*_KPC-2_-harboring IncFII plasmids (Tian et al., 2021, 2022).

The clinical scenario of in-host co-colonization and infection by distinct classical and hypervirulent CRKP clones represents a complex challenge that significantly complicates diagnosis and treatment strategies. In this study, from a long-term surveillance of ICU patients colonized or infected with carbapenem-non-susceptible bacteria, we identified distinct classical and hypervirulent CRKP clones from a single patient, including classical CRKP strains belonging to ST4496 and hv-CRKP strains classified as ST268-KL20. The ST268-KL20 clone has emerged as a particularly concerning hypervirulent carbapenem-resistant strain, with studies demonstrating that ST268-K20 *K. pneumoniae* isolates simultaneously harbor pLVPK-like virulence plasmids and *bla*_KPC-2_-encoding plasmids.

## Methods

### Strain and antimicrobial susceptibility

A long-term surveillance for colonization or infection with carbapenem-non-susceptible bacteria was conducted once per week on hospitalized patients after their admission. Each time, carbapenem-non-susceptible strains were screened separately from throat swabs and rectal swabs. Meanwhile, infections occurring during the screening period were monitored, including drug-resistant strains isolated from sterile body fluids such as blood and urine. Species identification was performed using matrix-assisted laser desorption ionization time-of-flight (MALDI-TOF) mass spectrometry (Bruker Daltonics, Bremen, Germany). The MICs of imipenem, meropenem, ertapenem, aztreonam, amikacin, colistin, tigecycline, and ceftazidime/avibactam were determined by broth microdilution. All sample screening, bacterial identification, and MIC determination were performed in the clinical microbiology laboratory. After isolation and identification, the bacterial strains were immediately preserved in glycerol broth at −80°C for long-term storage. The MIC results were interpreted according to the Clinical and Laboratory Standards Institute (CLSI) 2024 guidelines. Isolates were considered carbapenem resistant if the MICs of meropenem or imipenem were ≥4 mg/L or the MIC of ertapenem was ≥2 mg/L. *Escherichia coli* ATCC 25922 served as the quality control strain.

### Whole-genome sequencing and phylogenetic analysis

Genomic DNA was extracted using the QIAamp DNA Mini Kit (Qiagen, Hilden, Germany) according to the manufacturer’s instructions and subsequently sequenced on the Illumina HiSeq X Ten platform (Illumina, San Diego, CA, USA). The resulting short reads were *de novo* assembled using Shovill. One strain was selected for long-read sequencing on a MinION sequencer (Oxford Nanopore Technologies, Oxford, UK). A *de novo* hybrid assembly combining Illumina and Nanopore reads was performed using Unicycler v0.4.8 (Wick et al., 2017). A phylogenetic tree was constructed based on single-nucleotide polymorphism (SNP) differences using Snippy (https://github.com/tseemann/snippy).

### Sequence typing, serotyping, antimicrobial resistance genes, and virulence genes

Multilocus sequence typing (MLST) was performed according to the Pasteur scheme. Capsular typing was conducted using Kaptive (Wyres et al., 2016). Antimicrobial resistance genes were identified with ABRicate v0.8.13 (https://github.com/tseemann/abricate) using the ResFinder database (http://genomicepidemiology.org/) (Zankari et al., 2012). Bacterial virulence factors were identified via the Virulence Factor Database (VFDB, http://www.mgc.ac.cn/VFs/) (Liu et al., 2019), focusing mainly on siderophore systems including yersiniabactin (*ybtAEPQSTUX*), salmochelin (*iroBCDN*), and aerobactin (*iucABCDiutA*), as well as the genotoxin colibactin and the polysaccharide regulators *rmpA*/*rmpA2* genes (Russo et al., 2018; Turton et al., 2019; Lan et al., 2020).

### Plasmid manipulation and analysis

A conjugation assay was conducted to assess whether the plasmids carrying carbapenem resistance determinants were transferable. *Escherichia coli* J53 (sodium azide-resistant) served as the recipient strain, and conjugation experiments were performed using the filter mating method (Yang et al., 2021). Transconjugants were selected on Mueller–Hinton (MH) agar plates supplemented with meropenem (1 mg/L) and sodium azide (200 mg/L). Plasmid replicons were identified using PlasmidFinder v2.1 based on genome sequencing data (Carattoli et al., 2014). Circular maps for plasmid comparisons were generated with Proksee (Grant et al., 2023).

### Mouse infection model

We employed a mouse infection model to evaluate the pathogenicity of the bacterial strains, using female BALB/c mice, a classical inbred strain (Yan et al., 2021). Four groups of mice (10 mice per group) were established in this study, and each group was inoculated intraperitoneally with 5 × 10^7^ CFU of bacteria. The inoculation dose was determined based on the 48-h LD_50_ values from preliminary infection experiments with multiple *K. pneumoniae* strains, ensuring a sufficient pathogenic concentration after bacterial inoculation. Post-injection, the mice were monitored every 12 h for a total of 72 h, and mortality was recorded. Kaplan–Meier survival curves were plotted based on the mortality data, and statistical differences between the curves were analyzed using the Mantel–Cox test. A *P*-value of ≤0.05 was considered statistically significant.

## Results

### A series of CRKP strains identified from infection and colonization

**Figure 1:**
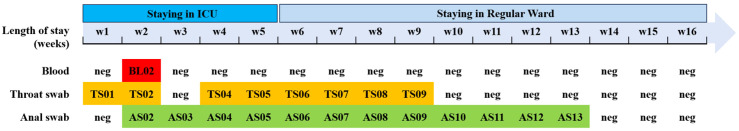
Ward admission timeline and detection times of positive strains from the blood, throat swabs, and rectal swabs in the hospitalized patient.

Through this long-term surveillance of ICU patients colonized or infected with carbapenem-non-susceptible bacteria, consecutively isolated resistant strains were identified. One hospitalized patient drew our particular attention, as CRKP strains were repeatedly isolated from multiple sample types over several weeks, including both colonization and infection sites. Therefore, we performed a longitudinal analysis of all CRKP isolates obtained from this patient. This patient was admitted due to severe pancreatitis and was hospitalized in the ICU (weeks 1–5) followed by a general ward stay (weeks 6–16). Screening for carbapenem-resistant strains began in the first week of admission and continued until the patient was discharged at week 16. Throat and rectal swabs were collected weekly for screening. Eight CRKP isolates were recovered from throat swabs during weeks 1, 2, 4/5, 6, 7, 8, and 9—four from the ICU stay and four from the general ward. These isolates were designated TS01, TS02, TS04, TS05, TS06, TS07, TS08, and TS09. Twelve CRKP isolates were obtained from rectal swabs during weeks 2–13—four during the ICU stay and eight during the general ward stay. These isolates were named AS02, AS03, AS04, AS05, AS06, AS07, AS08, AS09, AS10, AS11, AS12, and AS13. In addition, one CRKP strain was isolated from the patient’s blood during ICU admission in week 2 and was designated BL02. In total, 21 CRKP isolates were collected from this patient throughout the hospitalization period (**Figure 1**).

### Phylogenetic relationship among CRKP isolates

**Figure 2:**
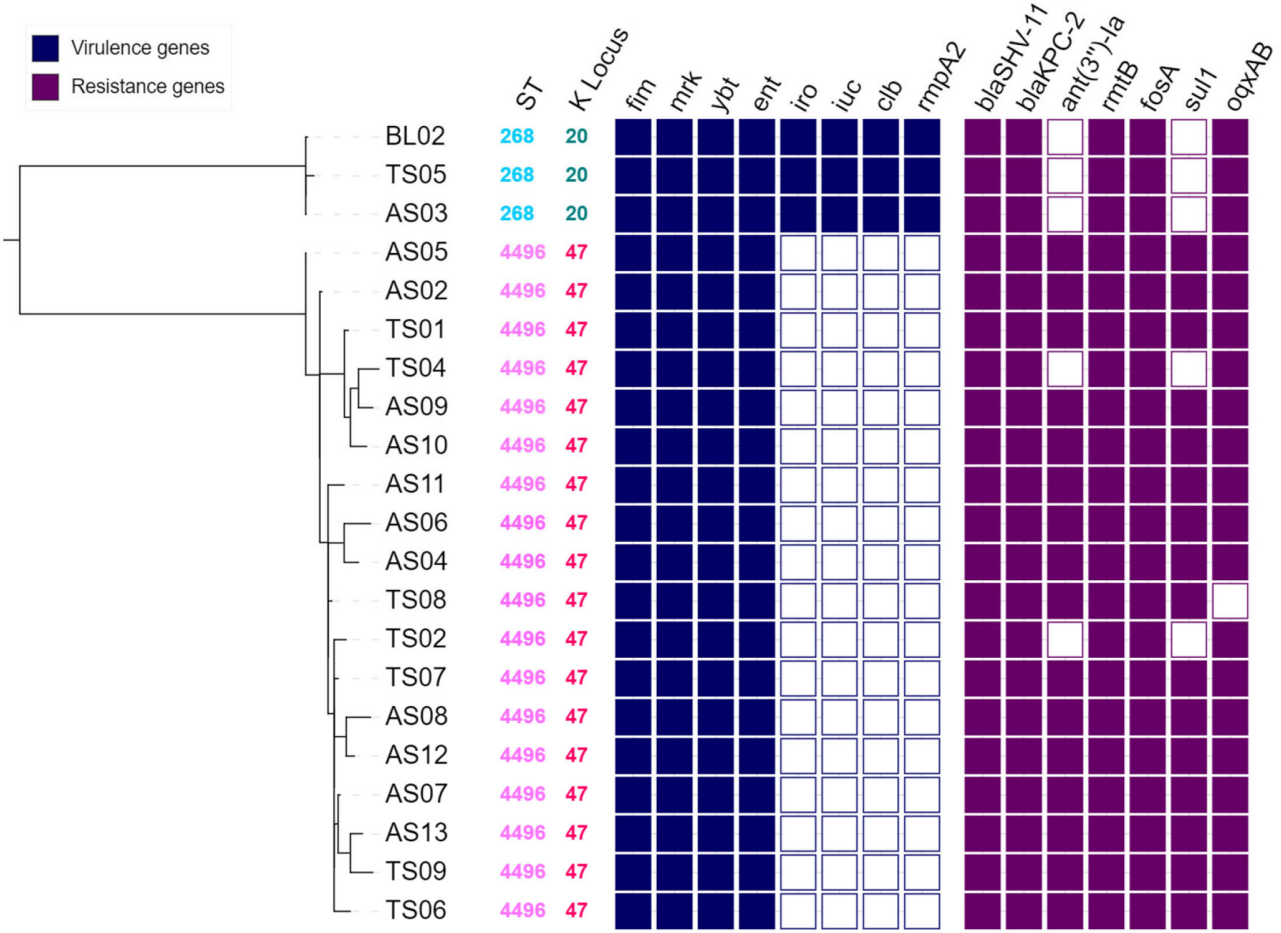
Phylogenetic tree of the 21 CRKP isolates identified through screening.

All 21 CRKP isolates collected from the patient underwent whole-genome sequencing using the Illumina platform, yielding draft genome assemblies. Additionally, the bloodstream infection (BSI) strain BL02 was sequenced using the Nanopore platform, and hybrid assembly enabled the reconstruction of its complete chromosomal and plasmid sequences. To clarify the clonal relationship among the isolates, all of them were subjected to sequence analysis and phylogenetic tree construction (**Figure 2**). Based on sequence typing, the isolates fell into two sequence types (STs): three isolates belonged to ST268, and the other 18 isolates were ST4496, which is part of clonal complex CC11 and differs from the epidemic clone ST11 by a single allelic mutation in the *mdh* locus. Specifically, the bloodstream isolate BL02 from week 2, the rectal isolate AS03 from week 3, and the throat isolate TS05 from week 5 were all ST268 clones. The remaining isolates belonged to the ST4496 clone. Within each of these two clones, the strains exhibited very close genetic relatedness, with SNP differences ranging from 1 to 5 SNPs among the ST268 isolates and 5 to 42 SNPs among the ST4496 isolates. In contrast, the genetic distance between the two STs was substantial, with more than 28,356 SNP differences separating them.

Each isolate on the phylogenetic tree is annotated with its MLST type and serotype, as well as the presence of antimicrobial resistance genes and virulence genes. Dark-colored boxes indicate gene presence, while white boxes indicate gene absence. The branch length between the two clonal clusters, ST268 and ST4496, has been shortened for visualization purposes.

### Multidrug-resistant profile of the serial isolates

All 21 K*. pneumoniae* isolates collected from the patient were classified as CRKP, thus exhibiting resistance to carbapenems and presenting a multidrug-resistant profile. However, the two distinct clones exhibited different levels of antimicrobial resistance, and we evaluated them based on MIC values and the distribution of resistance genes. Among the ST4496 clone, all 18 isolates had meropenem MICs exceeding 16 mg/L, ertapenem MICs over 8 mg/L, and imipenem MICs no lower than 16 mg/L. In contrast, the ST268 clone showed comparatively lower resistance to carbapenems. The three ST268 isolates all had imipenem MICs of 8 mg/L, while the meropenem MIC for the isolate TS05 was only 2 mg/L, falling into the intermediate susceptibility range; for this strain, the ertapenem MIC was merely 4 mg/L. Apart from carbapenems, all isolates exhibited high-level resistance to aztreonam and amikacin, with MICs exceeding 32 and 128 mg/L, respectively. Regarding several clinically important therapeutic options for CRKP infections, none of the isolates demonstrated resistance to polymyxin or tigecycline. However, for ceftazidime/avibactam, four isolates had MICs reaching 16 mg/L, indicating resistance (**Supplementary Table 1**).

Genomic analysis of acquired resistance genes revealed highly similar resistance gene profiles among the isolates. All strains harbored the *bla*_KPC-2_ gene, suggesting that production of KPC-2 carbapenemase was the primary mechanism conferring carbapenem resistance. Additionally, all strains carried the β-lactamase gene *bla*_SHV-11_, as well as the aminoglycoside resistance gene *rmtB* and the fosfomycin resistance gene *fosA*. Some differences were observed between the ST268 and ST4496 clones. For example, the ST268 isolates lacked the aminoglycoside-modifying enzyme gene *ant(3″)-Ia* and the sulfonamide resistance gene *sul1*, both of which were present in the majority of ST4496 isolates (17 out of 19).

### Virulent profile of the serial isolates

Next, we evaluated the virulent profile of the serial isolates. The capsular serotype (K locus) of *K. pneumoniae* is closely associated with its virulence and pathogenic potential. Among the 21 isolates collected from this patient, the capsular serotypes were consistent with their respective clonal types, dividing them into two groups: the three ST268 isolates were of the KL20 type, while the remaining ST4496 isolates belonged to the KL47 type. Regarding virulence gene profiles, in addition to common chromosomal genes present in all *K. pneumoniae* strains—such as fimbrial genes (*fim* and *mrk*), Enterobactin siderophore system, and Yersiniabactin—the ST268-KL20 strains also harbored Salmochelin, Aerobactin, the toxin Colibactin, and the hypermucoviscosity regulator gene *rmpA2*, demonstrating typical characteristics of hvKP.

**Figure 3:**
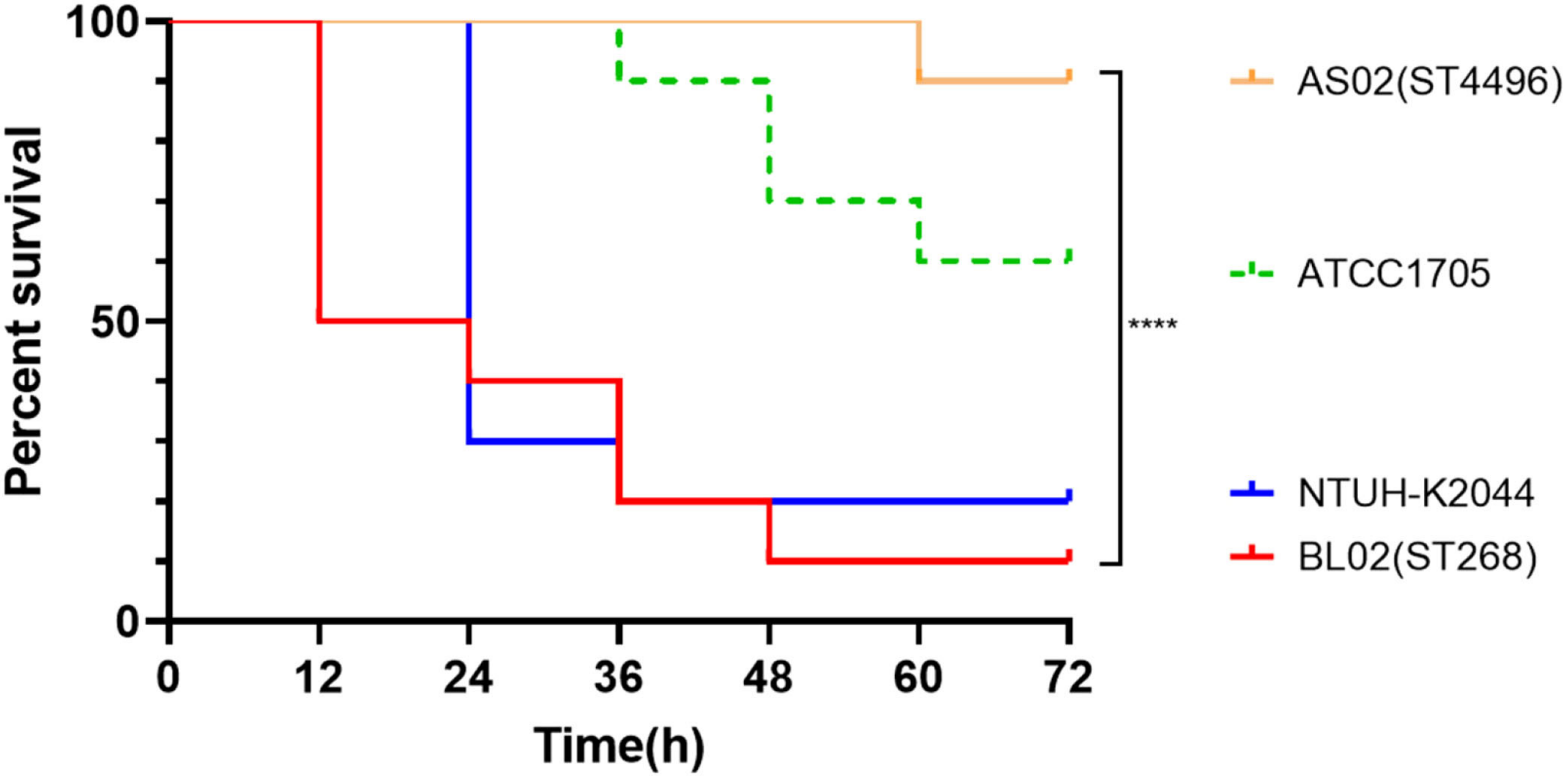
Survival curves and comparisons from the mouse infection model for the clinical representative strains BL02 (blood isolate), AS02 (intestinal isolate), Classic hvKP reference strain NTUH-K2044, and low-virulence reference strain ATCC 1705.

We evaluated the pathogenic potential of several representative isolates using a mouse infection model. The ST4496 representative strain, AS02, exhibited low lethality in mice, comparable to the low-virulence reference strain ATCC 1705, with the ST4496 clone displaying even lower virulence levels. In contrast, the representative ST268 isolate, the bloodstream-derived strain BL02, demonstrated high lethality in mice similar to the classic hypervirulent strain NTUH-K2044 (Wu et al., 2009), with the ST268 strain’s virulence being even more pronounced in some instances (**Figure 3**).

### Horizontal transfer of a *bla*_KPC-2_-encoding plasmid

**Figure 4:**
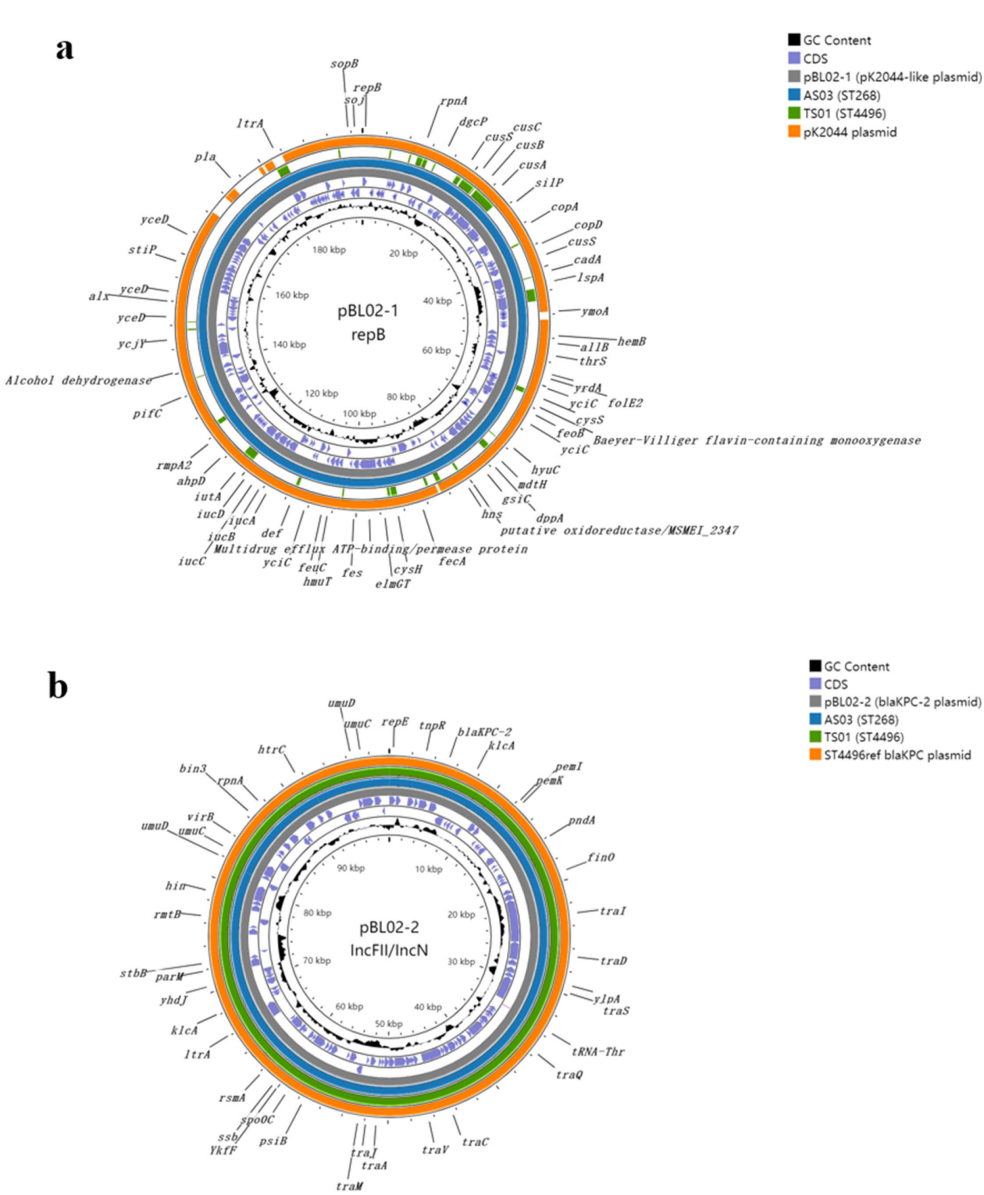
Circular comparison maps of *bla*_KPC-2_-harboring plasmids in representative strains. **(A)** From innermost to outermost rings: GC content; complete *bla*_KPC-2_-harboring plasmid from the bloodstream isolate BS02 with annotated coding sequences (CDSs); mapping of the ST268 clone AS03 draft assembly; mapping of the ST4496 clone TS01 draft assembly; reference *bla*_KPC-2_-harboring plasmid from a known ST4496 strain in the GenBank database. **(B)** From innermost to outermost rings: GC content; complete pK2044-like plasmid from the bloodstream isolate BL02 with annotated CDSs; mapping of the ST268 clone AS03 draft assembly; mapping of the ST4496 clone TS01 draft assembly; pK2044-like plasmid from the classic hypervirulent *K. pneumoniae* strain.

The complete genome sequence analysis provided insights into the potential horizontal gene transfer events between different clones. Thus, we performed hybrid sequencing combining second- and third-generation technologies on the ST268 strain BL02 to obtain its complete chromosomal and plasmid sequences. The results revealed that, in addition to a chromosome of 5.37 Mb, the strain BL02 carried three plasmids: pBL02-1 (197.894 kbp), pBL02-2 (99.768 kbp), and pBL02-3 (3.198 kbp). Comparison with known plasmid sequences in the GenBank database showed that plasmid pBL02–1 closely resembled the typical virulence plasmid pK2044 from the hypervirulent strain NTUH-K2044, differing only by an ~17-kbp deletion around the *rmpA* gene. However, pBL02–1 retained aerobactin and *rmpA2*, classifying it as a pK2044-like plasmid. Meanwhile, pBL02–2 was identified as a typical *bla*_KPC-2_-carrying plasmid, highly similar to *bla*_KPC-2_-encoding plasmids previously reported in ST4496 strains in the database. Mapping analyses were conducted using draft genome assemblies from other isolates in this study against pBL02–1 and pBL02-2 (**Figure 4**). The two other ST268 strains showed 100% alignment with both plasmids, suggesting they likely harbor identical plasmids and belong to the same clone. In contrast, all ST4496 isolates carried plasmids encoding *bla*_KPC-2_ (100% coverage) but showed less mapping to the pK2044-like plasmid. Conjugation experiments demonstrated that strains from both clones were capable of transferring the *bla*_KPC-2_-carrying plasmid to recipient strains. Altogether, these findings suggest that horizontal transfer of a *bla*_KPC-2_-encoding plasmid may have occurred between the two clonal lineages of CRKP isolates recovered from this patient.

## Discussion

Our study highlights the importance of monitoring intestinal and respiratory tract colonization in ICU patients. Continuous surveillance not only revealed persistent colonization by multidrug-resistant organisms (MDROs) but also provided evidence that colonizing bacteria can enter the bloodstream and cause infection (Sun et al., 2019; Du et al., 2023). In this case, during the patient’s 16-week hospital stay, their intestinal and respiratory tracts were colonized for most of the time by CRKP strains. Notably, two distinct CRKP clones were identified: one was the ST4496 clone belonging to the CG11 clonal complex—currently the most prevalent CRKP clonal type in Chinese clinical settings (Shi et al., 2024)—carrying a plasmid-encoded *bla*_KPC-2_ gene; the other was the ST268 clone, which not only carried a *bla*_KPC-2_-positive plasmid but also harbored a pK2044-like plasmid carrying the siderophore aerobactin and the hypermucoviscosity regulator gene *rmpA2*. This combination integrates carbapenem resistance with the hypervirulent phenotype characteristic of CR-hvKP (Liu et al., 2023). We found significant phenotypic and genotypic differences between the two colonizing CRKP clones. Importantly, the CR-hvKP clone was responsible for BSI, and the *bla*_KPC-2_-harboring plasmid it carried was likely acquired via horizontal transfer from the other CRKP clone.

The bloodstream isolate BL02 showed high genomic similarity, resistance profiles, and virulence characteristics closely matching those of the intestinal colonizer AS03 and the respiratory colonizer TS05, providing strong evidence for clonal dissemination from colonization to infection. Although the strains in this study were all isolated from a single patient, there have been many previous case reports describing the transition of hv-CRKP or CR-hvKP from colonization to infection. For example, in a large-scale molecular epidemiological investigation, it was reported that hv-CRKP strains carrying a positive type VI secretion system (T6SS) were more likely to undergo a transition from intestinal colonization to BSI (Zhao et al., 2025). Interestingly, these two ST268 colonizing strains were isolated in week 3 and week 5 after admission—both later than the bloodstream isolate detected in week 2. In fact, some studies suggest that there is no difference in patient outcomes depending on whether bacterial colonization or infection occurs first, as it is inherently difficult to distinguish whether a clinically isolated strain represents colonization or infection (Howard-Anderson et al., 2022). Nevertheless, in this study, we believe that colonization in the gut or respiratory tract occurred prior to the bacteria entering the bloodstream and causing BSI. If the frequency of colonization screening were increased, it might be possible to isolate the colonizing strain before the bloodstream isolate BL02 appeared. The respiratory colonizer TS01, detected in week 1, belonged instead to the ST4496 clone. These findings indicate that in the same patient, infection and colonization by the same bacterial species can involve phenotypically distinct clones. Different clones may “mask” each other, complicating clinical judgment and antimicrobial decision-making. For example, the ST4496 clone, with higher levels of carbapenem resistance and a higher isolation frequency, is more likely to draw clinicians’ attention. However, this clone generally has lower virulence and is less likely to cause severe BSIs—potentially distracting clinicians from recognizing the hidden, more virulent CR-hvKP ST268 clone capable of causing severe invasive disease.

ST4496 belongs to CG11, the most prevalent CRKP clonal complex in China, where ST11 is the dominant sequence type with serotypes mainly KL64 and KL47. Multiple studies have shown KL64 strains to be significantly more virulent than KL47 (Zhou et al., 2020). In our study, ST4496 strains belonged to KL47, exhibiting relatively weaker virulence, and have been reported as common colonizers in ICU environments and patients (Zhao et al., 2021). By contrast, the ST268-KL20 clone is a typical hvKP strain carrying the pK2044-like virulence plasmid and multiple key virulence factors (Shen et al., 2020; Goncalves et al., 2025). Mouse infection models confirmed its hypervirulence, even exceeding that of the classic hvKP strain NTUH-K2044. In a previous report, an outbreak in a neonatal intensive care unit in northern Portugal was caused by 30 KPC-3-producing ST268-KL20 isolates. The outbreak was rapidly identified using a newly developed real-time Fourier-transform infrared (FT-IR) spectroscopy workflow and contained within 23 days, underscoring its high pathogenic potential (Goncalves et al., 2025). Furthermore, ST268-KL20 clones have demonstrated diversity in both resistance and virulence levels. A study reported two ST268-KL20 strains: one was multidrug-resistant (MDR) and carried *bla*_KPC-2_, while the other was non-MDR and carbapenem-susceptible. The former CRKP strain was hypermucoviscous and exhibited high serum survival and virulence in the *Galleria mellonella* model, whereas the latter displayed significantly lower virulence (Shen et al., 2020).

In our study, plasmid-mediated transfer was confirmed between the ST268 and ST4496 clones. They shared an approximately 100-kb *bla*_KPC-2_-harboring plasmid encoding a conjugation transfer operon, and experiments demonstrated successful conjugative transfer. We infer that this plasmid was transferred from the original host ST4496 clone to the hvKP ST268 clone, transforming it into a CR-hvKP strain. By acquiring the *bla*_KPC-2_-harboring plasmid in addition to its original pK2044-like plasmid, the ST268-KL20 clone integrated multidrug resistance with hypervirulence, posing a substantial threat to clinical infection management.

Our study also has several limitations. First, the study is based on isolates from a single patient. Although the detailed longitudinal sampling is a strength, this limitation reduces the generalizability of the findings. Second, we only completed whole-genome and plasmid sequencing for a subset of isolates, and we inferred that horizontal transfer of the *bla*_KPC-2_-harboring plasmid occurred. In fact, the complete genomes of all isolates should be obtained to ensure the rigor of the conclusion regarding plasmid dissemination.

In summary, we identified co-colonization by two distinct CRKP clones and a case where an hvKP clone evolved into CR-hvKP through acquisition of a *bla*_KPC-2_-harboring plasmid from a co-colonizing clone. The presence of multiple genetically distinct CRKP clones within individual patients creates a scenario where different clones may provide mutual protection through various mechanisms, potentially leading to treatment failures if therapeutic regimens are based on testing of only one isolate. This phenomenon of multiclone infections increases the complexity of clinical infection diagnosis and treatment, as the different KPC-producing clones may exhibit varying responses to antimicrobial therapy while harboring homologous *bla*_KPC-2_-harboring plasmids (Han et al., 2024), thereby enhancing the clinical challenges in infection management. The coexistence of these two CRKP clones, each adapted to the same ecological niche but with unique traits, increases the complexity and diversity of the pathogenic bacterial population, enhancing its adaptability under diverse selective pressures and potentially complicating treatment decisions for clinical infections.

## Data Availability

The datasets presented in this study can be found in online repositories. The names of the repository/repositories and accession number(s) can be found in the article/**Supplementary Material**.
