# Supplementary material for: In-host co-colonization and bloodstream infection by distinct classical and hypervirulent CRKP clones harboring a homologous blaKPC-2-harboring plasmid
Source: Front Cell Infect Microbiol. 2025 Dec 19;15:1683743. doi: 10.3389/fcimb.2025.1683743 (PMC12757419; doi:10.3389/fcimb.2025.1683743)
Supplement: Supplementary file 1 [file Table1.docx]

Supplementary Table 1. MLST types, K locus, and MIC values for several key therapeutic agents in the clinical serial isolates

| **Strain** | **ST** | **K Locus** | **IMP** | **MEM** | **ETP** | **TGC** | **CST** | **ATM** | **AMK** | **CAZ-AVI** |
| --- | --- | --- | --- | --- | --- | --- | --- | --- | --- | --- |
| BL02 | ST268 | KL20 | 8 | 8 | >8 | 2 | 0.25 | >32 | >128 | 4 |
| TS05 | ST268 | KL20 | 8 | 2 | 4 | 0.25 | 0.25 | >32 | >128 | 2 |
| AS03 | ST268 | KL20 | 8 | 8 | >8 | 4 | 0.25 | >32 | >128 | 2 |
| TS01 | ST4496 | KL47 | 32 | >16 | >8 | 0.5 | 0.5/4 | >32 | >128 | 8 |
| TS02 | ST4496 | KL47 | 32 | >16 | >8 | 0.5 | 0.125 | >32 | >128 | 8 |
| TS04 | ST4496 | KL47 | 64 | >16 | >8 | 0.5 | 0.25 | >32 | >128 | 16 |
| TS06 | ST4496 | KL47 | 32 | >16 | >8 | 0.5 | 0.125 | >32 | >128 | 8 |
| TS07 | ST4496 | KL47 | 32 | >16 | >8 | 0.5 | 0.125 | >32 | >128 | 16 |
| TS08 | ST4496 | KL47 | 64 | >16 | >8 | 1 | 0.5/4 | >32 | >128 | 8 |
| TS09 | ST4496 | KL47 | 64 | >16 | >8 | 1 | 0.25 | >32 | >128 | 16 |
| AS02 | ST4496 | KL47 | 32 | >16 | >8 | 0.5 | 0.25 | >32 | >128 | 8 |
| AS04 | ST4496 | KL47 | 16 | >16 | >8 | 0.5 | 0.125 | >32 | >128 | 8 |
| AS05 | ST4496 | KL47 | 64 | >16 | >8 | 0.5 | 0.125 | >32 | >128 | 8 |
| AS06 | ST4496 | KL47 | 64 | >16 | >8 | 0.25 | 0.125 | >32 | >128 | 8 |
| AS07 | ST4496 | KL47 | 32 | >16 | >8 | 0.5 | 0.25 | >32 | >128 | 8 |
| AS08 | ST4496 | KL47 | 16 | >16 | >8 | 0.5 | 0.125 | >32 | >128 | 4 |
| AS09 | ST4496 | KL47 | 32 | >16 | >8 | 0.5 | 0.25 | >32 | >128 | 16 |
| AS10 | ST4496 | KL47 | 32 | >16 | >8 | 0.5 | 0.125/1 | >32 | >128 | 8 |
| AS11 | ST4496 | KL47 | 32 | >16 | >8 | 0.5 | >8 | >32 | >128 | 8 |
| AS12 | ST4496 | KL47 | 64 | >16 | >8 | 0.5 | 0.125 | >32 | >128 | 8 |
| AS13 | ST4496 | KL47 | 16 | >16 | >8 | 0.5 | 0.125 | >32 | >128 | 8 |
